# Distinct Contributions of Working Memory and Attentional Control to Sentence Comprehension in Noise in Persons With Stroke

**DOI:** 10.1044/2021_JSLHR-20-00694

**Published:** 2021-07-20

**Authors:** Megan C. Fitzhugh, Arianna N. LaCroix, Corianne Rogalsky

**Affiliations:** a Stevens Neuroimaging and Informatics Institute, University of Southern California, Los Angeles, CA; b College of Health Sciences, Midwestern University, Glendale, AZ; c College of Health Solutions, Arizona State University, Tempe

## Abstract

**Purpose:**

Sentence comprehension deficits are common following a left hemisphere stroke and have primarily been investigated under optimal listening conditions. However, ample work in neurotypical controls indicates that background noise affects sentence comprehension and the cognitive resources it engages. The purpose of this study was to examine how background noise affects sentence comprehension poststroke using both energetic and informational maskers. We further sought to identify whether sentence comprehension in noise abilities are related to poststroke cognitive abilities, specifically working memory and/or attentional control.

**Method:**

Twenty persons with chronic left hemisphere stroke completed a sentence–picture matching task where they listened to sentences presented in three types of maskers: multispeakers, broadband noise, and silence (control condition). Working memory, attentional control, and hearing thresholds were also assessed.

**Results:**

A repeated-measures analysis of variance identified participants to have the greatest difficulty with the multispeakers condition, followed by broadband noise and then silence. Regression analyses, after controlling for age and hearing ability, identified working memory as a significant predictor of listening engagement (i.e., mean reaction time) in broadband noise and multispeakers and attentional control as a significant predictor of informational masking effects (computed as a reaction time difference score where broadband noise is subtracted from multispeakers).

**Conclusions:**

The results from this study indicate that background noise impacts sentence comprehension abilities poststroke and that these difficulties may arise due to deficits in the cognitive resources supporting sentence comprehension and not other factors such as age or hearing. These findings also highlight a relationship between working memory abilities and sentence comprehension in background noise. We further suggest that attentional control abilities contribute to sentence comprehension by supporting the additional demands associated with informational masking.

**Supplemental Material:**

https://doi.org/10.23641/asha.14984511

Sentence comprehension deficits are common following a left perisylvian stroke. This work has historically focused on differences in comprehending simple and complex sentence structures (e.g., Dronkers et al., 2004; Rogalsky et al., 2018; Thothathiri et al., 2012) and largely concludes that sentence comprehension deficits for complex sentence structures arise following a left hemisphere stroke because of deficits in grammar knowledge (Grodzinsky, 1995) or comorbid deficits in the cognitive resources that support language, such as attention (Kuzmina & Weekes, 2017; LaCroix et al., 2019, 2020; Murray, 2012; Peristeri et al., 2019) and working memory (e.g., Caplan et al., 2013; LaCroix et al., 2019; Potagas et al., 2011). Since persons with aphasia (PWA) have been shown to have spared ability to make grammaticality judgements (Wulfeck, 1988), we focus on the relationship between cognition and sentence comprehension in this study. The current work linking attention and working memory to sentence comprehension deficits poststroke has primarily focused on sentences presented in optimal listening conditions (i.e., silence), with relatively few studies investigating sentence comprehension in noisy conditions (e.g., broadband noise and multispeaker backgrounds). This is despite many persons with stroke (PWS), including PWA, reporting difficulty comprehending speech in noisy environments (Bamiou et al., 2012; Blaettner et al., 1989; Rankin et al., 2014; Skelly, 1975). It, therefore, may be that deficits in the cognitive resources, which support complex sentence comprehension, also contribute to the difficulties PWS experience comprehending speech in noisy environments. Thus, research is needed to examine the extent to which background noise affects sentence comprehension in PWS and what factors contribute to this in order to better understand the functional communication difficulties PWS experience when processing sentences in real-life listening conditions.

Ample work in neurotypical adults demonstrates increased difficulty comprehending speech in background noise (e.g., Schneider et al., 2007). Two components of background noise, energetic and informational masking, pose unique challenges to speech comprehension (Brungart et al., 2001; Schneider et al., 2007). Energetic masking reflects acoustic interference of the target and masking signals and is often experimentally generated by adding broadband noise over target speech, whereas informational masking is the conflation of similar-sounding and/or misplaced attention toward target and masking signals and is often generated by adding single- or multispeaker samples over target speech (Arbogast et al., 2005; Darwin, 2008). In middle-age and older adults, the comprehension of speech in multispeaker maskers requires more cognitive resources than speech in the presence of energetic maskers alone (Helfer et al., 2017; Rajan & Cainer, 2008). Several studies also report that these masking effects engage working memory and attentional control resources in order to facilitate speech comprehension, particularly in older adults (Akeroyd, 2008; Desjardins & Doherty, 2013; Fitzhugh et al., 2020; Peelle, 2018).

The ease of language understanding (ELU) model provides a framework for understanding and investigating the relationship between working memory and attentional control and speech-in-noise comprehension (Rönnberg et al., 2008). The ELU model posits that working memory (i.e., the temporary storage and manipulation of information) is required to resolve discrepancies between an incoming speech signal and stored phonological representations of known words; such discrepancies may arise when listening to speech in any type of noisy environment. The model further describes that aspects of attentional control (specifically, the inhibition of distractors) are involved in correctly perceiving a target speaker among multiple, competing speakers (Rönnberg et al., 2013). These relationships can be further contextualized within the recently proposed model of listening engagement (MoLE; Herrmann & Johnsrude, 2020), which posits that the recruitment of cognitive resources during listening (a process the authors term *listening engagement*) is determined, in part, by the limits of these cognitive resources, such that as listening conditions become noisier, listening engagement increases up to its limit, after which the listener disengages (Herrmann & Johnsrude, 2020). Taken together, the ELU and MoLE models suggest that PWS with deficits in working memory will have increased listening engagement for sentences embedded in any background noise, while attentional control deficits will show increased listening engagement specifically for informational masking effects.

Studies of PWS (with and without aphasia) investigating auditory processing in noisy environments largely have focused on single words and indicate that speech-in-noise comprehension is impaired compared to when speech is presented in silence (Healy et al., 2007; Kittredge et al., 2006; Raymer et al., 2019; Winchester & Hartman, 1955). Notably, the greater impairment for speech in noise than in silence was not found to be related to age or hearing levels (Healy et al., 2007; Raymer et al., 2019; Winchester & Hartman, 1955). Few studies have investigated speech-in-noise comprehension using more complex language structures such as sentences. The few that do explore this area report findings similar to studies using single words: PWS, including PWA, demonstrate greater difficulty comprehending sentences in background noise (both energetic and information masking) compared to silence (Basili et al., 1980; Villard & Kidd, 2019). This difference is not due to impaired hearing thresholds or measures of global cognition, including attention, assessed by the Cognitive Linguistic Quick Test–Plus and the Map Search and Elevator Counting With Distraction subtests of the Test of Everyday Attention (Villard & Kidd, 2019). While Villard and Kidd (2019) reported investigating the role of attentional control in speech-in-noise processing, none of their attention tasks specifically measured this construct (Helm-Estabrooks, 2001; Robertson et al., 1994; Stewart & Amitay, 2015). Thus, the relationship between cognition and sentence comprehension in noise needs to be investigated in PWS using more sensitive assessments of the cognitive domains proposed by the ELU model (i.e., working memory and attentional control).

The purpose of this study was to examine the relationships between sentence comprehension in noise, using both energetic and informational maskers, and cognition, specifically attentional control and verbal working memory (hereto referred to as *working memory*). Sentence comprehension was assessed using a sentence–picture matching task. Working memory and attentional control were measured using well-established tasks that are sensitive to each construct: the Wechsler Adult Intelligence Scale–Fourth Edition Working Memory Index (WAIS-IV WMI; Wechsler, 2008) and a color–word Stroop task (MacLeod, 1991). Our participant sample includes PWS with and without aphasia, as it is well documented that both groups have sentence comprehension difficulties under optimal listening conditions (e.g., Dronkers et al., 2004; Rogalsky et al., 2018; Thothathiri et al., 2012) and exhibit similar deficits in working memory and attention (Bonini & Radanovic, 2015; Lee & Pyun, 2014). Thus, PWS may have greater individual differences in cognition than neurotypical controls, which allows us to better probe the associations between sentence comprehension and cognition proposed in the ELU and MoLE models. Our specific interest in the impact of background noise on sentence comprehension prompted us to assess sentence comprehension using simple sentence structures so as to avoid the potential language confounds associated with complex sentence comprehension already established in our participant sample. We further focus our analyses on reaction times (RTs), not accuracy, as they are a commonly used psychometric indicator of cognitive processing, including listening engagement (Gatehouse & Gordon, 1990), and are sensitive to differences between PWS without an aphasia diagnosis and neurotypical controls on sentence comprehension measures similar to the one used in this study (Salis et al., 2021).

For the task overall, we hypothesized that participants would demonstrate longer RTs when sentences were presented in multispeakers compared to broadband noise and in broadband noise compared to silence. Following the terminology of the MoLE, longer RTs will be indicators of increased listening engagement and reflect greater recruitment of cognitive resources during listening. Based on the framework provided by the ELU and MoLE models, we hypothesized that poorer working memory would be associated with greater listening engagement in all background noise conditions and for speech in energetic masking (which we computed via a RT difference score): Those with reduced working memory resource limits will likely need to maintain and rehearse misheard speech information for a longer period before making a response. We also hypothesized that greater listening engagement in response to speech in informational masking (also computed via an RT difference score) would be associated with poorer attentional control performance: Those with poorer attentional control resources will need more time to discern and attend to the target speaker while ignoring background speakers.

## Method

### Participants

Participants were recruited as part of a larger study investigating the role of cognition in language comprehension more broadly. The included participants represent a subset of participants from the larger study who completed the sentence–picture matching task in background noise and also had the cognitive measures of interest. Participants were 20 adults (12 women) who experienced a single left hemisphere cerebral stroke at least 6 months prior to testing. Participants ranged in age from 28 to 78 years (*M =* 55.45, *SD* = 14.47) and had between 12 and 20 years of education (*M =* 15.90, *SD* = 2.24). All participants were premorbidly right-handed, native speakers of American English and reported no history of neurological or psychiatric disorders or head trauma prior to their stroke. Of the 20 stroke participants, 14 had a diagnosis of aphasia confirmed by the Boston Diagnostic Aphasia Examination–Third Edition (BDAE-3; Goodglass et al., 2000); each participant's aphasia classification is reported in Table 1 *.* To isolate the effects of background noise on sentence comprehension from potential language deficits, participants needed to have above chance performance (> .50 proportion correct) for the sentences presented in silence in order to be included in the study; all tested participants met this inclusion criteria (see Table 1). All participants were compensated monetarily for their participation. All procedures were approved by the Arizona State University Institutional Review Board.

**Table 1.** Participant demographics.

Participant	Gender	Age	Months poststroke	Years of education	BDAE-3 single-word reading comprehension	BDAE-3 auditory single word comprehension	Sentences in silence accuracy (proportion correct)	Aphasia diagnosis	WAIS-IV WMI point estimate	Stroop task point estimate
PWS 1	F	57	77	18	15/15	16/16	1.0	None	−1.2	−1.4
PWS 2	F	48	110	19	15/15	16/16	1.0	Broca's	−2.4^a^	−0.3
PWS 3	M	60	138	14	15/15	14/16	.80	Broca's	−4.5^a^	−0.4
PWS 4	F	75	179	16	15/15	15/16	.80	Broca's	−2.6^a^	−2.3^b^
PWS 5	F	73	53	16	15/15	16/16	1.0	Anomic	−1.9	−2.0
PWS 6	M	78	12	16	15/15	15/16	1.0	None	0.2	1.6
PWS 7	M	78	58	18	15/15	16/16	1.0	None	1.5	0.4
PWS 8	F	43	29	14	15/15	15/16	1.0	Broca's	−3.2^a^	2.2^a^
PWS 9	F	46	79	14	15/15	15/16	.70	Broca's	−3.5^a^	1.9
PWS 10	M	70	50	16	15/15	16/16	1.0	None	1.1	0.1
PWS 11	F	34	174	14	15/15	16/16	.90	None	−3.7^a^	0.6
PWS 12	F	40	63	20	12/15	16/16	1.0	Broca's	−0.6	−1.3
PWS 13	M	28	20	13	15/15	15/16	1.0	Anomic	−3.5^a^	0.6
PWS 14	F	59	110	15	15/15	16/16	.90	Anomic	−2.9^a^	7.0^a^
PWS 15	F	41	72	17	15/15	15/16	1.0	Broca's	−3.5^a^	−1.2
PWS 16	M	57	13	16	15/15	16/16	1.0	Broca's	−2.9^a^	−1.0
PWS 17	F	54	45	14	12/15	16/16	.90	Broca's	−3.5^a^	1.7
PWS 18	F	57	25	12	15/15	16/16	1.0	None	−2.4^a^	0.9
PWS 19	M	61	20	20	15/15	16/16	.90	Conduction	−2.2	1.6
PWS 20	M	50	233	16	6/15	14/16	.70	Broca's	−4.1^a^	−0.9

*Note.* BDAE-3 = Boston Diagnostic Aphasia Examination–Third Edition; WAIS-IV WMI = Wechsler Adult Intelligence Scale–Fourth Edition Working Memory Index; PWS = person with stroke; F = female; M = male.

a Point estimate of effect size is significant at *p* < .05, with PWS performing worse than controls.

b Point estimate of effect size is significant at *p* < .05, with PWS performing better than controls.

### Hearing

**Figure 1.**
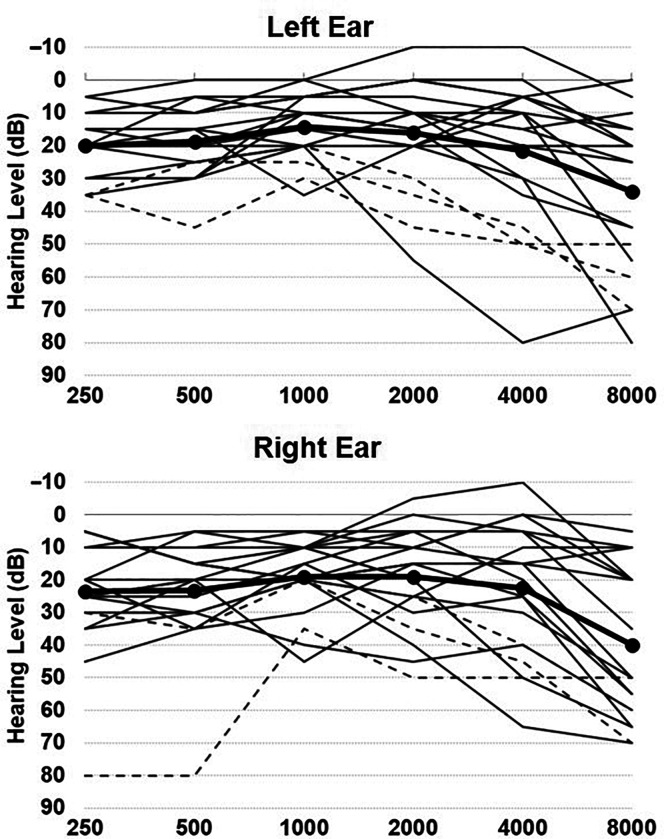
Hearing thresholds for left (top) and right (bottom) ear assessed by pure-tone audiometry. Individual participant thresholds are shown with solid lines, except thresholds for participants who wear hearing aids are shown with dashed lines. Group average thresholds are shown with the bolded lines.

Hearing acuity was assessed by pure-tone audiometry using a GSI 18 Audiometer and supra-aural headphones in a quiet room using a pulsed tone and a two-down, one-up procedure in steps of 5 dB for each correctly and incorrectly detected tone. Hearing acuity was summarized as the pure-tone average across 500–4000 Hz in both ears. Participants' pure-tone averages ranged from −1.88 to 48.13 dB (*M* = 19.28, *SD* = 12.19; see Figure 1). Three participants (PWS 3, 4, and 6) wore hearing aids during the sentence comprehension and cognitive tasks, but not during the hearing screening.

### Sentence Comprehension

Participants completed 60 trials^1^ of a sentence–picture matching task in which they were presented with 30 sentences with a simple canonical, subject–verb–object word order (e.g., The boy who is red is kissing the girl) and 30 sentences with a complex noncanonical subject–object–verb word order (e.g., The boy who the girl is kissed by is red). The noncanonical sentences were collected as part of a separate study and are excluded here as our primary interest lies in understanding the effects of background noise separate from any sentence comprehension difficulties the participants may have. Each sentence consisted of 10 syllables and contained two nouns (girl and boy), one of seven verbs (hug, push, kiss, pull, kick, wash, and chase), and one of three color adjectives (blue, green, and red). Thematic role assignment, verb, and adjective use were balanced across all sentences.

Sentences ranged in duration from 2.39 to 2.89 s (*M =* 2.60, *SD =* .10) and were presented in three different background maskers (10 trials per condition): multispeakers, broadband noise, and silence. The multispeaker background sample consisted of four speakers (two male and two female) reading sentences with unrelated content aloud. The long-term average spectrum of the multispeaker background sample was estimated via fast Fourier transform and a Hamming window function and applied to a sample of white noise to generate the broadband noise sample. All background noise conditions began 500 ms prior to the onset of the target sentence and stopped 500 ms after the offset of the sentence. The signal-to-noise ratio of the target sentence to background noise sample was + 2 dB. All sentences were recorded by a single male speaker in standard American English in Audacity 2.2.1 sound editing software (https://audacityteam.org/).

**Figure 2.**
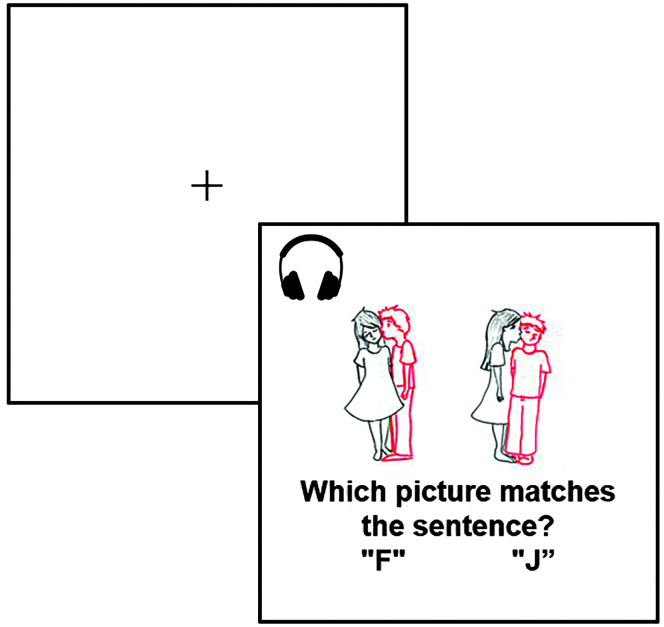
Visual schematic of one trial for the sentence–picture matching task.

The computerized sentence–picture matching task began with a fixation cross for 1,000 ms, followed by the simultaneous presentation of the auditory sentence and visual picture stimuli (see Figure 2). Sentences were presented randomly without replacement per participant. A picture pair was presented, with a target picture that matched the sentence and a foil picture that contained a different action verb or a reversal of agent and patient. The pictures remained on the screen until a response was made. Responses were given using two keyboard buttons corresponding to the left or right picture. Thus, chance performance was defined as .50 proportion correct or below. Following a response, trials ended with a fixation cross for 1,000 ms. Five null trials were randomly presented with a fixation cross but no auditory stimuli for 5,000 ms; no responses were required, and these trials were presented to be compatible with future functional magnetic resonance imaging studies. Participants were given both verbal and written instructions. Participants also completed five practice trials containing examples of all background maskers and were allowed to adjust the volume to a comfortable listening level. Participants were instructed to select the matching picture as accurately and quickly as possible. E-Prime Standard 2.0 software (Psychology Software Tools,) was used to deliver the task.

### Cognition

We assessed working memory (i.e., ability to temporarily store and manipulate information) using the WAIS-IV WMI (Wechsler, 2008). The index is derived of scaled scores from the Digit Span (forward, backward, and sequencing) and the Arithmetic subtests. For the Digit Span subtest, participants were presented with a sequence of numbers of increasing length and asked to repeat the sequence in the same order it was presented, in reverse order of presentation, and in ascending order. For the Arithmetic subtest, participants were auditorily presented with math problems of increasing complexity and given 30 s to solve each problem. The first five math problems contained picture supports. For both Working Memory subtests, we provided participants with a visual number line so that they could point to their response rather than verbalize it, thereby reducing language production demands; no participant elected to use this option. We measured verbal working memory as it is often more impaired in PWA than nonverbal working memory (e.g., Christensen & Wright, 2010) and is frequently associated with sentence comprehension deficits in PWA and neurotypical controls (Caspari et al., 1998; LaCroix et al., 2019; Newman et al., 2013; Rogalsky et al., 2008).

Attentional control, the ability to inhibit distracting information while focusing on the target stimulus, is commonly measured in PWA using the color–word Stroop task (Green et al., 2010; Kuzmina & Weekes, 2017; LaCroix et al., 2019; Pompon et al., 2015). Here, we used a computerized version of the color–word Stroop task in which participants saw one of eight words (color words: red, blue, green, and yellow; neutral words: small, solid, sleep, and start) presented horizontally in the center of the screen in one of four different ink colors (red, blue, green, and yellow). Single-word reading was evaluated as part of the BDAE-3; 17 of 20 participants performed at ceiling (see Table 1), suggesting that single-word reading difficulties likely did not interfere with color–word Stroop task performance. To complete the color–word Stroop task, participants had to inhibit reading the printed word and instead press the button corresponding to the color the word was printed in (e.g., for the word “blue” printed in red ink, the correct response would be “red”). Participants were presented with 24 congruent trials, 24 incongruent trials, and 32 neutral trials for a total of 80 trials.^2^ An RT difference score between mean RT for incongruent and neutral trials was computed, reflecting interference effects and the ability of attentional control resources to inhibit off-task information (MacLeod, 1991).

### Statistical Analysis

Accuracy and RT (in milliseconds) were recorded for each trial of the sentence–picture matching and Stroop tasks. For analyses regarding accuracy, all trials in a particular condition were included for each participant. For the RT analyses, average RT was computed for each participant using correct responses only and excluding RTs greater than 2.5 *SD*s from each participants' mean across all task conditions. This procedure is a well-established approach in psycholinguistic research (Baayen & Milin, 2010; Lachaud & Renaud, 2011; Ratcliff, 1993) and was determined a priori because PWS demonstrate abnormal online processing patterns for incorrect responses (Caplan et al., 2007; Dickey et al., 2007; Hanne et al., 2011). This procedure was therefore applied to ensure the process of interest is being captured and not other extraneous factors such as brief distractions or button press mistakes. Consistent with this procedure, 11.1% (errors: 9.8%; outliers: 1.3%) of the data were excluded from the sentence picture matching task (see Supplemental Material S1 for individual participant data), and 5% (errors: 5%; outliers: 0%) of data were excluded from the Stroop task.

#### Sentence Comprehension

We used logistic regression to determine the main effects of masker (silence, broadband noise, or multispeakers) on accuracy in the sentence–picture matching task. Logistic regressions were conducted in SAS software, Version 9.4. Similarly, a one-way repeated-measures analysis of variance (ANOVA) was computed to determine the effects of masker within mean RT from the sentence–picture matching task using SPSS Version 25.0 (IBM Corp.). The assumption of normality of the RT data was assessed using Shapiro–Wilk tests and a visual inspection of Q-Q plots. Significance was defined as *p* < .05, two-tailed. Post hoc comparisons were corrected for multiple comparisons using the Benjamini–Hochberg (BH) false discovery rate (FDR) procedure (Benjamini & Hochberg, 1995).

#### Cognitive Measures Predicting RT of Background Noise

Five hierarchical multiple regression models were used to investigate the relationship between cognition and RT (i.e., listening engagement) to sentences in noise in PWS. The regression models were computed in SPSS Version 25.0 (IBM Corp.) and followed standard practices regarding treatment of covariates and thresholding (Feise, 2002; Perneger, 1998; Rothman, 1990). Three of the dependent variables in the five regression models were mean RT from sentences presented in (a) multispeakers, (b) broadband noise, and (c) silence. It has been shown that speech maskers create a combination of energetic and informational masking effects (Rosen et al., 2013). Therefore, we computed RT difference scores within each participant to represent the unique effects of informational and energetic masking (Fitzhugh et al., 2020). These additional dependent variables were (d) the RT difference score subtracting broadband noise from multispeakers, which approximately isolates the effects of informational masking, and (e) the RT difference score subtracting silence from broadband noise, which approximately isolates the effects of energetic masking. In all five models, the independent variables were working memory and attentional control derived from the WAIS-IV WMI and Stroop task, respectively. Age and hearing thresholds were included as covariates in all models. Model significance was defined as *p* < .05, two-tailed, with BH FDR correction for multiple comparisons.

To further characterize each participant's cognitive abilities within our sample of PWS, we first used the SPSS “Explore” procedure to determine the potential effects of outliers within the independent variables via box plots. Two participants were identified as outliers, one for working memory (PWS 7, performing significantly better than the other participants) and one for attentional control (PWS 14, performing significantly worse than the other participants). We also quantified impairment on both the working memory and attentional control measures for each participant using single-case Bayesian hypothesis tests (Crawford et al., 2010; Crawford & Garthwaite, 2007). This approach allowed us to calculate the probability that a given participant's score on each task was impaired compared to a control group. The control group consisted of unpublished data from 20 neurotypical adults who were matched on age (*M =* 51.40, *SD* = 12.82 years), *t*(38) = 0.94, *p* = .36; education (*M* = 15.9, *SD =* 2.17 years), *t*(38) = 1.00, *p* = .32; and gender (14 women), *χ* ^2^(1) = 3.64, *p* = .06, and completed the same cognitive assessments (WAIS-IV WMI: *M =* 108.15, *SD =* 13.06; Stroop: *M* = 60.07, *SD =* 55.02 ms) as part of another study in our laboratory. The output of this analysis is a standard score (point estimate of effect size), estimating the difference between the participant's score and the control group's mean. A participant was classified as “impaired” on a particular task if the participant's score differed from the control group's mean at a significance level of *p* < .05 (Crawford & Garthwaite, 2007; Crawford et al., 2010).

## Results

### Sentence Comprehension

#### Accuracy

**Figure 3.**
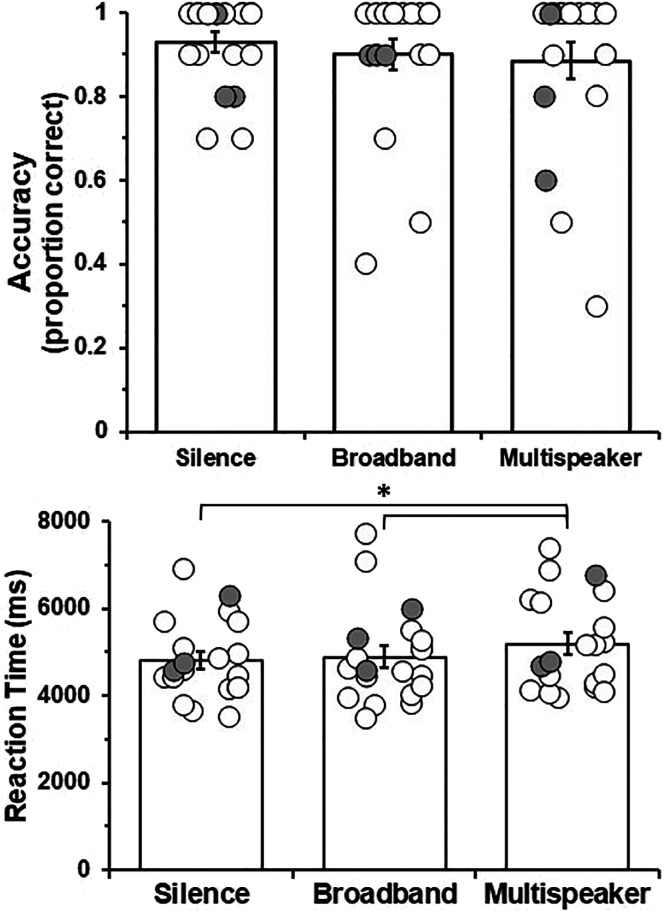
Mean and individual accuracy (top) and reaction time (bottom) for the sentence–picture matching task. Gray circles represent the three participants with hearing aids. Error bars represent ±1 *SEM*. *BH FDR *p* < .05. BH FDR = Benjamini–Hochberg false discovery rate.

Mean and individual sentence–picture matching task accuracy for each condition are depicted in Figure 3. The main effect of masker for accuracy was not significant, *χ* ^2^(2) = 1.92, *p* = .38.

#### RT

Mean and individual sentence–picture matching task RT are depicted in Figure 3. The distribution of participants' mean RT for sentences in silence did not significantly deviate from a normal distribution, *W*(20) = .94, *p* = .27; however, mean RT for sentences in broadband noise and multispeakers did significantly deviate from a normal distribution: broadband noise, *W*(20) = .90, *p* = .04; multispeakers, *W*(20) = .90, *p* = .049. Visual inspection of Q-Q plots of mean RT for each condition confirmed a nonnormal distribution of the data. Therefore, to improve normality, mean RTs for all conditions of the sentence comprehension task were log transformed and then entered into the one-way repeated-measures ANOVA (Pek et al., 2017).

The main effect of masker was significant, *F*(2, 38) = 4.03, *p* = .026. Pairwise comparisons between each level of masker were computed using paired-samples *t* tests with correction for multiple comparisons. Sentences in multispeakers had significantly longer RTs (*M* = 5,199.9 ms, *SD* = 1,078.6, without log transformation) compared to sentences in silence (*M* = 4,815.9 ms, *SD* = 898.5, without log transformation), *t*(19) = 2.71, FDR *p* = .04, and compared to sentences in broadband noise (*M* = 4,895.0 ms, *SD* = 1,070.6 without log transformation), *t*(19) = 2.44, FDR *p* = .04. RTs between sentences in broadband noise and sentences in silence did not significantly differ, *t*(19) = 0.38, *p* = .71.

*Single-case comparisons.* The single-case Bayesian hypothesis tests demonstrated that 13 out of our 20 participants had impaired working memory and two out of 20 had impaired attentional control compared to a matched control group (see Table 1). Of the 13 participants with impaired working memory, all but two (PWS 11 and 18) were diagnosed with aphasia. Both participants with impaired attentional control (PWS 8 and 14) were diagnosed with aphasia. An exploratory, post hoc independent-samples *t* test between those with impaired (*n* = 13) versus unimpaired (*n* = 7) working memory in each sentence condition indicated that those with impaired working memory (*M* = 5,521.3 ms, *SD* = 1,159.5, without log transformation) had greater listening engagement to sentences in multispeakers, *t*(17.65) = −2.2, *p* = .04 (Levene's test indicated unequal variances [*F* = 4.81, *p* = .04], so degrees of freedom were adjusted from 18), compared to those with unimpaired working memory (*M* = 4,603.1 ms, *SD* = 596.5 without log transformation)*,* but the two groups did not differ on the broadband noise, *t*(18) = −1.26, *p* = .22), or silence conditions, *t*(18) = −1.44, *p* = .17. The post hoc comparison for impaired versus unimpaired attentional control could not be computed due to only two PWS having impaired attentional control.

### Cognitive Measures Predicting RT of Background Noise

**Figure 4.**
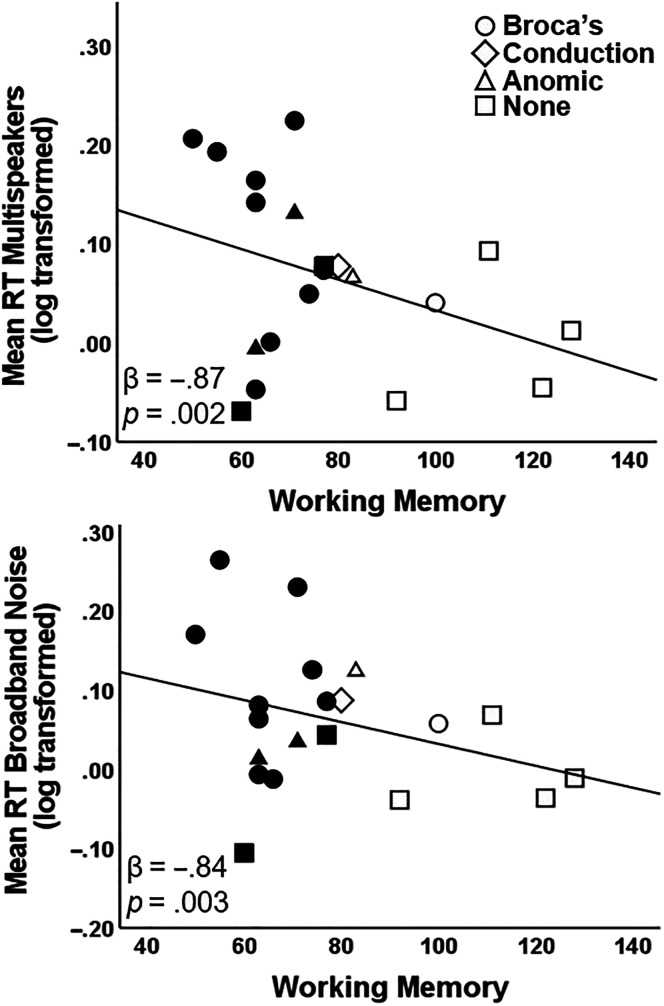
Partial regression plots between working memory and mean reaction times (RT) for sentences in multispeakers (top) and in broadband noise (bottom), controlling for age and pure-tone average. Participants' aphasia diagnosis is indicated by the different symbols. Black filled symbols represent participants whose working memory scores were significantly impaired compared to a matched control group. The mean RTs for both multispeakers and broadband noise were log transformed.

In each regression model, age and pure-tone average were included as covariates, and working memory and attentional control derived from the WAIS-IV WMI and Stroop task, respectively, were the independent variables. The overall multiple regression model predicting mean RT for sentences in multispeakers was significant, *F*(4, 15) = 3.89, *R* ^2^ = .51, FDR *p* = .04. Working memory was the only significant predictor (β = −.87, *p* = .002); poorer working memory was associated with longer RTs (i.e., increased listening engagement; see Table 2 and Figure 4). Similarly, the regression model predicting mean RT for sentences in broadband noise was also significant, *F*(4, 15) = 3.71, *R* ^2^ = .50, FDR *p* = .03, with working memory being the only significant predictor; participants with poorer working memory demonstrated longer RTs (i.e., increased listening engagement; β = −.84, *p* = .003; see Table 2 and Figure 4). The model predicting sentences in silence using mean RT was not significant, *F*(4, 15) = 2.43, *R* ^2^ = .39, FDR *p* = .09.

**Table 2.** Multiple regression models predicting mean reaction time (RT) and RT difference scores for the stroke group.

Mean RT: sentences in multispeakers			
Predictors	β	*t*	*p*
Age	.42	1.57	.14
Hearing status	.24	0.92	.37
Working memory	−.87	−3.75	.002*
Attentional control	.20	1.09	.30
**Mean RT: sentences in broadband noise**			
**Predictors**	**β**	***t***	***p***
Age	.41	1.52	.15
Hearing status	.26	0.99	.34
Working memory	−.84	−3.60	.003*
Attentional control	−.24	−1.40	.21
**RT difference: effect of informational masking**			
**Predictors**	**β**	***t***	***p***
Age	−.03	0.12	.91
Hearing status	.00	0.001	.99
Working memory	−.08	−0.42	.68
Attentional control	.76	4.60	<.001*

*Note.* Only regressions for which the model was significant are presented.

* *p <* .05.

The RT difference scores of the effect of informational masking (i.e., RT for sentences in multispeakers − sentences in broadband noise) did not significantly deviate from a normal distribution, *W*(20) = .96, *p* = .51; however, the distribution of RT difference scores of the effect of energetic masking (i.e., RT difference score for sentences in broadband noise − sentences in silence) did significantly deviate from a normal distribution, *W*(20) = .83, *p* < .01; this was confirmed by visual inspection of the Q-Q plot. Therefore, the RT difference scores of energetic masking were transformed by adding a constant to the scores (due to the presence of negative values) and then log transformed.

**Figure 5.**
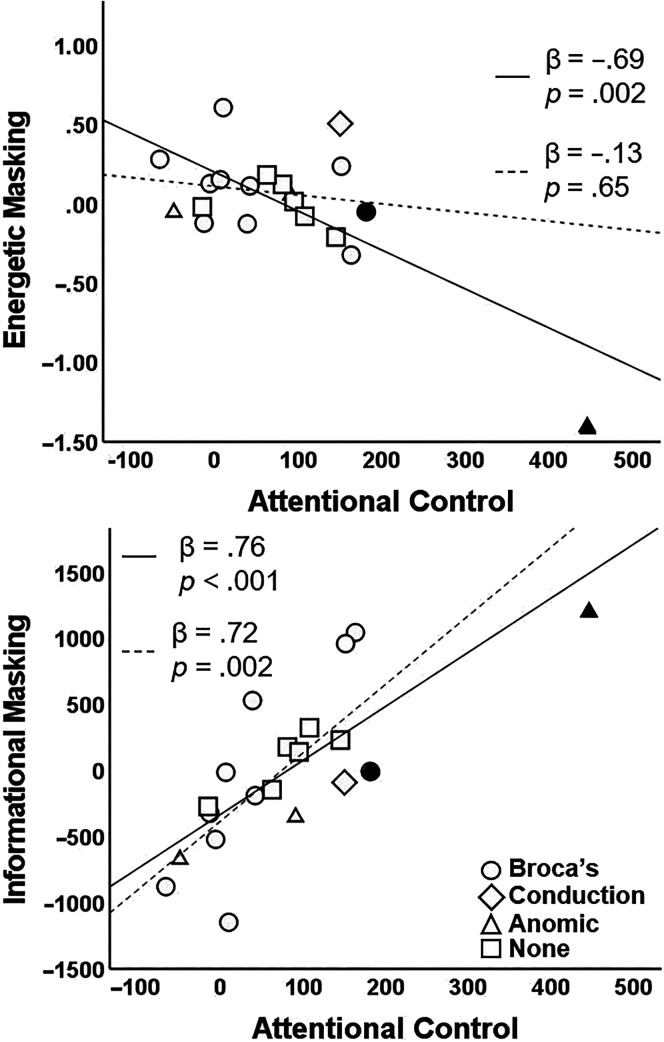
Partial regression plots between attentional control and reaction time difference scores representing effects of informational masking (top) and energetic masking (bottom), controlling for age and pure-tone average. Participants' aphasia diagnosis is indicated by the different symbols. Black filled symbols reflect participants whose attentional control scores were significantly impaired compared to a matched control group. Dashed lines reflect the regression with the outlying participant removed. The difference score for energetic masking was log transformed.

The model predicting the effect of energetic masking was significant, *F*(4, 15) = 3.99, *R* ^2^ = .52, FDR *p* = .03, with attentional control as the only significant predictor (β = −.69, *p =* .002; see Figure 5). However, it is noteworthy that this relationship was driven by one participant (PWS 14), and the model was no longer significant once this outlying participant was removed from the analysis, *F*(4, 14) = 0.14, *R* ^2^ = .04, *p* = .97 (attentional control β = −.13, *p =* .65). The multiple regression model predicting the effect of informational masking was significant, *F*(4, 15) = 5.52, *R* ^2^ = .60, FDR *p* = .03, with attentional control being the only significant predictor (β = .76, *p <* .001). In other words, participants with poorer attentional control demonstrated a greater increase in RT (i.e., greater listening engagement) in the presence of informational masking (see Table 2 and Figure 5). This model remained significant after removing the participant (PWS 14) with the outlying attentional control score, *F*(4, 14) = 3.63, *R* ^2^ = .51, *p* = .03 (attentional control β = .72, *p =* .002).^3^ Please see Supplemental Material S2 for the regression models in PWS with a formal aphasia diagnosis.

## Discussion

Sentence comprehension deficits following a left hemisphere stroke are thought to stem, in part, from a reduction in the availability or efficacy of cognitive resources that support sentence comprehension. However, research linking cognition and sentence comprehension deficits post left hemisphere stroke has primarily focused on sentences presented in optimal listening conditions (silence), despite everyday conversations often occurring in noisy environments. The purpose of this study was to identify the relationship between working memory and attentional control and comprehending sentences in noisy listening conditions in PWS.

Our findings demonstrate no differences in accuracy between each background noise condition, suggesting that participants could sufficiently hear the target sentence and that their poststroke sentence comprehension abilities did not confound their listening engagement. Similarly, their high accuracy indicates that deficits in grammar knowledge also likely did not contribute to their difficulty comprehending sentences in noise. In contrast to accuracy, we did find that PWS, on average, were slower when sentences were presented in multispeakers compared to sentences in broadband noise and silence. Listening engagement and masking effects may therefore be better captured using RT, instead of accuracy, as they reflect individual differences in effort and provide new insight into the resources involved in speech-in-noise comprehension. We interpret the variability in RT within each condition to reflect differences in listening engagement during sentence comprehension in different forms of noise and suggest that PWS may exert the greatest listening engagement specifically in multispeaker environments, similar to what is seen in neurotypical adults (Helfer et al., 2017; Rajan & Cainer, 2008). This increased listening engagement likely reflects the increased recruitment of cognitive resources that allow PWS to maintain relatively high comprehension accuracy. However, since sentence loudness was not adjusted based on each participant's pure-tone average, it is still possible that participant's ability to hear the stimuli may factor into the observed RT variability. Despite this potential limitation, the high levels of accuracy across all three listening conditions in our study suggest that participant's hearing did not detrimentally affect their comprehension. Additionally, pure-tone average was included as a covariate in all regression analyses predicting RT and was not a significant predictor in any model. Nonetheless, future studies should consider presenting stimuli at a consistent level or adjusted for each participant's hearing deficits.

The variability of listening engagement observed across PWS was predicted by cognitive ability; however, the cognitive predictor differed by background masking effect. As hypothesized, listening engagement to sentences in the presence of any background noise was predicted by working memory ability. Conversely, we found that listening engagement in response to informational masking (i.e., RT difference score between sentences in multispeakers and broadband noise), but not the effects of energetic masking (i.e., RT difference score between sentences in broadband noise compared to silence), was related to attentional control. Together, these findings align with the ELU model (Rönnberg et al., 2008) and provide evidence for the relative recruitment of specific cognitive resources in PWS during sentence comprehension, depending upon the specific properties of the background noise.

Working memory has previously been associated with comprehending speech in background noise in neurotypical controls (see Dryden et al., 2017, for meta-analysis); however, this relationship is greatly understudied in PWS. Here, we extend this literature, finding that PWS with lower working memory limits exhibit greater listening engagement in response to speech in any background noise (i.e., broadband noise and multispeakers tested in this study). In other words, even with relatively simple canonical sentences, which when presented in silence elicit no significant relationship with cognitive measures, the addition of either type of background noise increases listening engagement by specifically recruiting working memory.

Our single-case analyses revealed that those with impaired working memory abilities had greater listening engagement for sentences in multispeakers (mean RT) compared to those with unimpaired working memory. While the sample size of each group is insufficient to report separate regressions for each group, examination of the regression beta weights in the models for working memory and mean RT for the broadband noise and multispeakers conditions (see Supplemental Material S3) reveals the same negative relationship between working memory and sentence comprehension in noise for the unimpaired group, as observed in the entire sample. This suggests that working memory, if it is relatively intact, can contribute to the ability to comprehend sentences in background noise, perhaps by allowing one to hold and rehearse the target speech information as the sentence unfolds. However, for the impaired working memory group, the relationship is certainly less clear and more variable, suggesting that individuals with impaired working memory may be using some other strategy or resource during comprehension. It is possible that PWS with impaired working memory may engage their attentional control abilities more to support sentence comprehension in background noise. This possibility is supported by our single-case analysis, which revealed that 11 out of 13 PWS from the impaired working memory group had unimpaired attentional control performance compared to the matched control group. Thus, it could be that their attentional control abilities are recruited to help accommodate the added difficulty associated with informational masking, but not energetic masking. However, future work is needed to investigate the strategies or resources that may contribute to the variability in sentence comprehension performance in stroke survivors with impaired working memory.

In a related study of informational and energetic masking effects on sentence comprehension in PWA, Villard and Kidd (2019) examined the effects of masking on performance using a different type of sentence comprehension task. While Villard and Kidd classify all their participants as having aphasia, five of their 12 participants had a Western Aphasia Battery–Revised (WAB-R) Aphasia Quotient greater than 93.8, indicating that these participants are not classified as having aphasia according to the WAB-R's published norms (Kertesz, 2007). In their study, participants listened to sentences and then were asked to point to a picture of the sentence's object from a four-picture array; the same four pictures were displayed for every trial. They found that their “PWA” group performed similarly to controls on the energetic masking conditions but exhibited reduced performance compared to control participants on the informational masking condition, suggesting that PWA are specifically susceptible to the effects of informational masking and that this susceptibility is not due to age, hearing thresholds, language ability (measured by the WAB-R), or attention (measured by the Cognitive Linguistic Quick Test–Plus and select subtests of the Test of Everyday Attention). However, the authors propose that the standardized tests used in their study may not have been sensitive to the specific cognitive resources recruited during speech-in-noise processing. For example, the Cognitive Linguistic Quick Test–Plus's Attention domain does not specifically measure attentional control (Helm-Estabrooks, 2001). Similarly, the Test of Everday Attention has been shown to be a general measure of attention, while tasks like the Stroop and flanker are thought to capture inhibition of distractors (i.e., attentional control; Stewart & Amitay, 2015). Furthermore, the two specific subtests used by Villard and Kidd, Map Search and Elevator Counting With Distraction, are more strongly associated with working memory and sustained attention rather than inhibition (Robertson et al., 1994).

The lack of a relationship between measures of attention and speech comprehension in noise found by Villard and Kidd (2019) could also be explained by the relatively simple sentence constructions and comprehension task. Each sentence used by Villard and Kidd contained three words: a subject, a verb, and an object (e.g., Nina wants spoons). Comprehension was measured using a prompt, such as “What does Nina want?” and a four-picture array from which to select the correct response. Thus, it could be that the three-word sentences were not sufficient to tax attentional control or that the comprehension task itself, which required the participants to attend to only the last word in order to complete the task, was similarly too simple to recruit attentional control resources despite the presence of informational and energetic masking effects. While there are inherent differences between our study and that of Villard and Kidd (e.g., task design and cognitive assessments), our results largely align with their conclusions. We find PWS demonstrate greater listening engagement for informational masking compared to energetic masking and silence, suggesting that this relationship is not unique to PWS or PWA with clinically significant language impairments. However, our use of potentially more sensitive assessments of sentence comprehension and attentional control, specifically the ability to inhibit distracting information assessed using the Stroop task, allows us to suggest that listening engagement for informational masking is related to individual differences in attentional control in PWS.

Language demands likely confound the measures of cognition obtained in this study. While we made every attempt to reduce language production demands during the cognitive tasks (e.g., providing a number line), we were not able to make the same level of accommodations for language comprehension. We did follow the WAIS-IV administration protocol, which allowed for one repetition of each arithmetic question if requested. However, language comprehension abilities are still likely influencing the participant's working memory score, particularly since aphasia diagnosis is often associated with poorer performance on cognitive tasks, such as the backward digit span, compared to those with left hemisphere strokes but no aphasia diagnosis (Bonini & Radanovic, 2015; Lee & Pyun, 2014). Nonetheless, our findings regarding the ELU and MoLE models in PWS is similar to what has been previously reported in controls (Desjardins & Doherty, 2013; Fitzhugh et al., 2020; Schneider et al., 2007). We therefore do not believe that aphasia diagnosis is solely driving our participant's cognitive deficits and subsequently our results. Nevertheless, future work is needed to further quantify these relationships in PWS and PWA separately, using both verbal and nonverbal measures of cognition to better mitigate potential language confounds.

To summarize, this study examined how background noise affects sentence comprehension in PWS. We further explored the association between listening engagement to sentences in noise and cognition. We report that listening engagement, measured using mean RTs, increased when sentences were presented in background noise, regardless of type, and that this increase was largely associated with working memory abilities. Critically, in response to informational masking only, listening engagement differences were associated with attentional control resources, in that PWS with poorer attentional control demonstrated longer RTs (i.e., greater listening engagement). Overall, these findings align with the ELU and MoLE models and demonstrate that cognition supports sentence comprehension in noise in PWS. This work further highlights the need to examine communication in more ecologically valid settings poststroke.

## Supplementary Material

10.1044/2021_JSLHR-20-00694SMS1 Supplemental Material S1 Individual participant data.

10.1044/2021_JSLHR-20-00694SMS2 Supplemental Material S2 Multiple regression models predicting mean RT and RT difference scores for persons with aphasia only.

10.1044/2021_JSLHR-20-00694SMS3 Supplemental Material S3 Multiple regression models predicting mean RT and RT difference scores for participants with impaired and unimpaired working memory.

## Footnotes

1 One person with stroke only completed 30 trials due to technical difficulties (see Supplemental Material S1).

2 Three participants completed a previous version of the Stroop task that was identical in terms of experimental parameters (e.g., stimuli and timing) and only differed on the number of trials (instead of 80 trials, they completed 40 trials: 12 congruent, 12 incongruent, and 16 neutral trials).

3 Logistic regressions, ANOVAs, and linear regression models do not substantially change if the three PWS with hearing aids are removed.
