# Supplementary material for: Distinct Contributions of Working Memory and Attentional Control to Sentence Comprehension in Noise in Persons With Stroke
Source: J Speech Lang Hear Res. 2021 Jul 20;64(8):3230–41. doi: 10.1044/2021_JSLHR-20-00694 (PMC8740654; doi:10.1044/2021_JSLHR-20-00694)
Supplement: Supplemental Material S1 [file JSLHR-64-3230-s001.pdf]

Supplemental Material S1. Individual participant data.

	Number of trials in analysis (# excluded due to inaccuracy, due to RT outlier)		
**Participant**	**Multispeakers**	**Broadband Noise**	**Silence**
PWS 1	10 (0, 0)	10 (0, 0)	10 (0, 0)
PWS 2	9 (1, 0)	10 (0, 0)	10 (0, 0)
PWS 3	6 (4, 0)	8 (1, 1)	8 (2, 0)
PWS 4	8 (1, 1)	9 (1, 0)	7 (2, 1)
PWS 5	10 (0, 0)	10 (0, 0)	10 (0, 0)
PWS 6	10 (0, 0)	9 (1, 0)	10 (0, 0)
PWS 7	10 (0, 0)	10 (0, 0)	10 (0, 0)
PWS 8	8 (2, 0)	9 (1, 0)	9 (0, 1)
PWS 9	3 (7, 0)	5 (5, 0)	7 (3, 0)
PWS 10	10 (0, 0)	10 (0, 0)	10 (0, 0)
PWS 11	10 (0, 0)	10 (0, 0)	9 (1, 0)
PWS 12	10 (0, 0)	9 (0, 1)	10 (0, 0)
PWS 13*	5 (0, 0)	5 (0, 0)	5 (0, 0)
PWS 14	10 (0, 0)	10 (0, 0)	9 (1, 0)
PWS 15	10 (0, 0)	9 (1, 0)	9 (0, 1)
PWS 16	10 (0, 0)	9 (1, 0)	10 (0, 0)
PWS 17	8 (2, 0)	4 (6, 0)	8 (1, 1)
PWS 18	10 (0, 0)	10 (0, 0)	10 (0, 0)
PWS 19	9 (1, 0)	10 (0, 0)	9 (1, 0)
PWS 20	5 (5, 0)	7 (3, 0)	7 (3, 0)

*Data collection error resulting in 15 total trials instead of the normal 30 total trials.
