# Supplementary material for: Distinct Contributions of Working Memory and Attentional Control to Sentence Comprehension in Noise in Persons With Stroke
Source: J Speech Lang Hear Res. 2021 Jul 20;64(8):3230–41. doi: 10.1044/2021_JSLHR-20-00694 (PMC8740654; doi:10.1044/2021_JSLHR-20-00694)
Supplement: Supplemental Material S2 [file JSLHR-64-3230-s002.pdf]

Supplemental Material S2. Multiple regression models predicting mean RT and RT difference scores for persons with aphasia only.

**Mean RT: Sentences in Multispeakers**			
Overall model: $F(4, 9) = 1.17$, $p = .39$, $R^2 = .34$			
Predictors	β	*t*	*p*
Age	0.38	1.25	.24
Hearing Status	0.07	0.19	.86
Working Memory	–0.42	–1.50	.17
Attentional Control	0.22	0.73	.49
**Mean RT: Sentences in Broadband Noise**			
Overall model: $F(4, 9) = 1.43$, $p = .30$, $R^2 = .39$			
Predictors	β	*t*	*p*
Age	0.44	1.48	.17
Hearing Status	–0.06	–0.19	.86
Working Memory	–0.34	–1.26	.24
Attentional Control	–0.39	–1.33	.22
**Mean RT: Sentences in Silence**			
Overall model: $F(4, 9) = 0.59$, $p = .68$, $R^2 = .21$			
Predictors	β	*t*	*p*
Age	0.34	1.00	.34
Hearing Status	0.18	0.47	.65
Working Memory	–0.23	–0.74	.48
Attentional Control	0.07	0.21	.84
**RT Difference: Effect of Informational Masking**			
Overall model: $F(4, 9) = 4.08$, $p = .04$, $R^2 = .64$			
Predictors	β	*t*	*p*
Age	–0.03	–0.14	.89
Hearing Status	0.20	0.78	.46
Working Memory	–0.14	–0.67	.52
Attentional Control	0.84	3.77	.004
**RT Difference: Effect of Energetic Masking†**			
Overall model: $F(4, 8) = 3.67$, $p = .62$, $R^2 = .049$			
Predictors	β	*t*	*p*
Age	–0.10	–0.45	.67
Hearing Status	–0.14	–0.52	.62
Working Memory	–0.13	–0.61	.56
Attentional Control	–0.84	–3.64	.005

†Model is not significant with attentional control outlier removed.
