# Supplementary material for: Distinct Contributions of Working Memory and Attentional Control to Sentence Comprehension in Noise in Persons With Stroke
Source: J Speech Lang Hear Res. 2021 Jul 20;64(8):3230–41. doi: 10.1044/2021_JSLHR-20-00694 (PMC8740654; doi:10.1044/2021_JSLHR-20-00694)
Supplement: Supplemental Material S3 [file JSLHR-64-3230-s003.pdf]

Supplemental material, Fitzhugh et al., "Distinct Contributions of Working Memory and Attentional Control to Sentence Comprehension in Noise in Persons With Stroke," *JSLHR*, https://doi.org/10.1044/2021_JSLHR-20-00694

Supplemental Material S3. Multiple regression models predicting mean RT and RT difference scores for participants with impaired and unimpaired working memory.

	**Unimpaired**			**Impaired**		
**Mean RT: Sentences in Multispeakers**	Overall model: $F(4, 6) = 5.48$, $p = .16$, $R^2 = .92$			Overall model: $F(4, 8) = 1.18$, $p = .39$, $R^2 = .37$		
Predictors	β	*t*	*p*	β	*t*	*p*
Age	0.17	0.72	.60	0.55	0.99	.35
Hearing Status	0.48	1.71	.23	0.06	0.11	.92
Working Memory	–1.21	–4.36	.049	–0.38	–1.29	.24
Attentional Control	0.40	1.63	.25	0.22	0.73	.49
**Mean RT: Sentences in Broadband Noise**	Overall model: $F(4, 6) = 3.42$, $p = .24$, $R^2 = .87$			Overall model: $F(4, 8) = 1.43$, $p = .31$, $R^2 = .42$		
Predictors	β	*t*	*p*	β	*t*	*p*
Age	0.13	0.44	.71	0.47	0.88	.41
Hearing Status	0.55	1.57	.26	0.10	0.19	.86
Working Memory	–1.23	–3.61	.07	–0.29	–1.02	.34
Attentional Control	0.03	0.11	.93	–0.26	–0.88	.41
**Mean RT: Sentences in Silence**	Overall model: $F(4, 6) = 7.95$, $p = .12$, $R^2 = .94$			Overall model: $F(4, 8) = 0.79$, $p = .56$, $R^2 = .28$		
Predictors	β	*t*	*p*	β	*t*	*p*
Age	0.25	1.22	.35	0.39	0.66	.53
Hearing Status	0.73	3.07	.09	0.19	0.32	.76
Working Memory	–1.14	–4.90	.04	–0.19	–0.59	.57
Attentional Control	–0.49	–2.58	.12	0.18	0.55	.60
**RT Difference: Effect of Informational Masking**	Overall model: $F(4, 6) = 2.98$, $p = .27$, $R^2 = .86$			Overall model: $F(4, 8) = 2.89$, $p = .09$, $R^2 = .59$		
Predictors	β	*t*	*p*	β	*t*	*p*
Age	–0.04	–0.13	.91	0.08	0.19	.86
Hearing Status	–0.35	–0.95	.44	0.02	0.04	.97
Working Memory	0.50	1.37	.31	–0.12	–0.48	.65
Attentional Control	0.84	2.85	.10	0.78	3.16	.01
**RT Difference: Effect of Energetic Masking**	Overall model: $F(4, 6) = 0.69$, $p = .66$, $R^2 = .58$			Overall model†: $F(4, 8) = 8.91$, $p < .01$, $R^2 = .82$		
Predictors	β	*t*	*p*	β	*t*	*p*
Age	–0.34	–0.62	.60	–0.42	–1.41	.20
Hearing Status	–0.36	–0.57	.63	0.34	1.13	.29
Working Memory	0.28	0.46	.69	–0.04	–0.25	.81
Attentional Control	0.80	1.58	.26	–0.81	–4.85	.001

†Model is not significant with attentional control outlier removed
